# Predictors of early androgen deprivation treatment failure in prostate cancer with bone metastases

**DOI:** 10.1002/cam4.594

**Published:** 2016-01-14

**Authors:** Eberhard Varenhorst, Rami Klaff, Anders Berglund, Per Olov Hedlund, Gabriel Sandblom, the Scandinavian Prostate Cancer Group (SPCG) Trial No. 5

**Affiliations:** ^1^ Department of Urology and Department of Clinical and Experimental MedicineLinköping UniversityLinköpingSweden; ^2^ EpiStatUppsalaSweden; ^3^ Department of UrologyKarolinska InstituteSolnaSweden; ^4^ Department of Clinical Sciences, Intervention and Technology (CLINTEC)Karolinska Hospital HuddingeHuddingeSweden

**Keywords:** Androgen deprivation treatment, bone metastases, clinical predictors, early failure, prostate cancer

## Abstract

Approximately 15% of men with hormone naïve metastatic prostate cancer primarily fail to respond to androgen deprivation treatment (ADT). The reason why the response to ADT differs in this subgroup of men with prostate cancer remains unclear. The aim of this study was to describe the characteristics of these men and to thereby define predictors of early ADT failure in prostate cancer patients with bone metastases. The study was based on 915 men from the prospective randomized multicenter trial (no. 5) conducted by the Scandinavian Prostate Cancer Group comparing parenteral estrogen with total androgen blockade. Early ADT failure was defined as death from metastatic prostate cancer within 12 months after the start of ADT. Multivariate logistic regression models were applied to identify clinical predictors of early ADT failure. Ninety‐four (10.3%) men were primarily nonresponders to ADT. Independent predictors of early ADT failure were poor Eastern Cooperative Oncology Group performance status (PS), analgesic consumption, low hemoglobin, and high Soloway score (extent of disease observed on the scan), in where patients with poor PS and/or high analgesic consumption had a threefold risk of early ADT failure. Not significantly factors related to early ADT failure were age, treatment, cardiovascular comorbidity, T category, grade of malignancy, serum estrogen level, and SHBG at enrolment. We analyzed characteristics of a subgroup of patients who primarily failed to respond to ADT. Four independent clinical predictors of early ADT failure could be defined, and men exhibiting these features should be considered for an alternative treatment.

## Introduction

Androgen deprivation treatment (ADT) has been the gold standard in treatment of prostate cancer with distant metastases for more than half a century 1, 2, 3. However, the response to ADT varies, and approximately 15% of men with metastatic disease primarily fail to respond to ADT 1, 4, 5, 6, 7, 8, 9, 10, 11, 12. Not only did Huggins and coworkers describe the significant improvement in the clinical condition of patients with metastatic prostate cancer treated with ADT, they also defined a new disease state of early ADT‐refractory prostate cancer, that is, early ADT failure 1. In their series of 21 consecutive patients “a noticeable improvement occurred in the clinical status for all but three patients.”

In the vast majority of cases ADT leads to symptomatic relief, regression of metastases, and a fall in serum Prostate Specific Antigen (PSA). With optimal management, median survival time for men with metastatic disease is now 30–49 months 13 and survival longer than 10 years occasionally occurs 14. With time, however, the disease ceases to respond to hormone manipulation, becomes castration resistant, and the patient eventually dies of cancer progression unless death due to an unrelated cause intervenes. The secondary ADT failure (castration resistance) of metastatic prostate cancer after long‐term ADT has received increasing attention over the last two decades. The reason for this is that new hormonal and chemotherapeutic methods have been introduced and the mechanisms behind the development of castration resistance have been extensively explored 11, 15, 16, 17. As yet there are no predictors of early ADT failure. Results of previous studies on clinical associations between androgen receptors and treatment response in prostate cancer have been questioned 4, 18. Theories of the present time include both androgen receptor dependent and androgen receptor independent mechanisms 19, 20. Biochemical and histochemical assays have failed to predict primary response to ADT 21, 22, 23. Much focus has been placed on serum testosterone and other sex hormone levels and their relation to prostate cancer; but the results have been inconclusive 24, 25, 26. Although certain gene alterations or signatures have prognostic significance in primary prostate cancer, the clinical impact of primary prostate cancer genomic events has yet to be seen 17, 27, 28, 29. Models using molecular profiles of the primary tumor were not superior to models using clinical variables only to predict disease progress 30. The aim of this study was to analyze early ADT failure and to define clinical predictors associated with short survival due to early ADT failure in prostate cancer patients with bone metastases.

The 915 patients in this study were taken from a prospective noninferiority clinical trial (No. 5) conducted by the Scandinavian Prostate Cancer Group.

## Methods and Patients

### Clinical characteristics

This study was based on a Phase III study on 915 men with hormone‐naive metastatic prostate cancer from 61 centers in Denmark, Finland, Iceland, Norway and Sweden. Patients were included between December 1992 and June 1997 31, 32. To be eligible, patients were required to have skeletal metastases (M1) and an Eastern Cooperative Oncology Group (ECOG) performance status (PS) of 0–2; 0 = denotes fully active; 1 = restricted in strenuous activity but ambulatory; 2 = ambulatory and capable of self‐care but unable to work). The primary objective of the trial was to determine whether or not overall‐ and cause‐specific survival following high‐dose parenteral estrogen therapy was inferior to that following Total Androgen Blockade (TAB). The extent of the primary tumor was determined using digital rectal examination according to the TNM classification from 1987 33 and cytological or histological specimens graded according to the World Health Organization (WHO) system 34. PSA, Alkaline Phosphatase (ALP), testosterone and hemoglobin levels were determined by routine laboratory testing before the start of treatment. Serum estrogen and Sex Hormone‐Binding Globulin (SHBG) levels were optional.

Skeletal involvement was assessed using bone scans supplemented by X‐ray when necessary. The extent of skeletal metastases was calculated according to a modified Soloway score: 1 = the total area of hot spots less than three lumbar vertebral bodies; 2 = total area of hot spots larger than of score 1, but <75% of the total scan; and 3 ≥ 75% of the total scan or super scan 35. The presence of nonregional lymph nodes and soft tissue metastases was not investigated by CT or MRT scan.

### Treatment

The patients were randomized to TAB or treatment with polyestradiol phosphate (240 mg) given by intramuscular injection every 2 weeks for 8 weeks and monthly thereafter. TAB consisted of orchiectomy or medical castration with the LHRH agonist triptorelin, according to clinician and patient preference, combined with 250 mg flutamide taken orally three times daily. In the TAB group, 298 patients chose orchiectomy, and 159 men received medical castration by monthly injection of the LHRH agonist triptorelin. No significant differences in survival between the two treatment groups were seen 31.

### Ethics

The study was performed in accordance with the recommendations of the Helsinki Declaration and approved by the ethics committees of all centers partaking in the study. Patients were given verbal and written information, and gave their informed consent to be included in the study.

### Statistical methods

**Figure 1:**
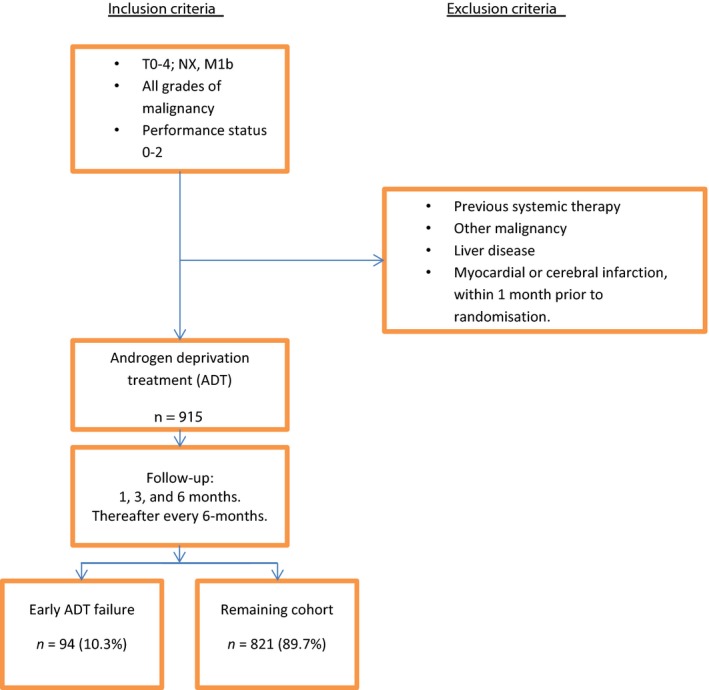
Flowchart showing study group assembly and follow‐up of the Scandinavian Prostate Cancer Group (SPCG) No. 5 Trial on androgen deprivation treatment (ADT) in men with prostate cancer and bone metastases. Early ADT failure was defined as disease‐specific death within 12 months after start of treatment.

In this study, early ADT failure was defined as a patient dying of prostate cancer within 12 months after start of treatment. The cohort of 915 patients was then divided into two groups: cases that were defined as having an early ADT failure (*n* = 94) and those in the remaining cohort (*n* = 821) (Fig. 1).

Differences in the characteristics of the early ADT failure group and those in the remaining cohort were tested using the chi‐squared test based on age at enrolment, cancer‐related pain, treatment, cardiovascular comorbidity, ECOG PS, analgesic consumption, grade of malignancy, T category, PSA level, Soloway score, hemoglobin, SHBG, estradiol, and testosterone levels. A univariate logistic regression model with odds ratio (OR) and corresponding 95% confidence interval (CI) was used to estimate the likelihood of early ADT failure for each variable. In order to identify independent predictors of early ADT failure, a multivariate logistic regression model was then constructed using all variables that were statistically significant in the univariate analyses. We applied three different criteria for early ADT failure. In a first scenario analysis, we defined primary ADT as death of any cause within 12 months (*N* = 137). In the second, we applied the same definition, but excluded 44 men who died of other causes them prostate cancer within 12 months from the study group, leaving 871 men for analyses. In the final scenario, we included all men who died of prostate cancer, regardless of time since randomization (*N* = 661 patients) but excluded those who died of other causes. For each scenario, we repeated all the univariate and multivariate logistic regression analyses. The study group assembly of the first scenario is shown in Figure 1. All *P*‐values were two‐sided and statistical significance was considered at *P* < 0.05. All analyses were performed using the R version 3.1.1 (Vienna, Austria).

## Results

Baseline demographic and clinical characteristics for the 915 patients enrolled in the study by the early ADT failure group and the remaining cohort are presented in Table 1.

**Table 1:** Demographic and clinical characteristics by the primary androgen deprivation treatment ADT failure group and the remaining cohort

	Primary ADT failure		Remaining cohort		*P*‐value	Total	
	*n*	%	*n*	%		*n*	%
All cases	94	100.0	821	100.0	–	915	100.0
Age, years							
<65	10	10.6	123	15.0		133	14.5
65–74	41	43.6	393	47.9		434	47.4
≥75	43	45.7	305	37.1	0.217	348	38.0
Initial treatment							
Polyestradiol phosphate	44	46.8	414	50.4		458	50.1
TAB	50	53.2	407	49.6	0,579	457	49.9
Cardiovascular comorbidity							
None	80	86.0	731	90.0		811	89.6
Present	13	14.0	81	10.0	0.354	94	10.4
Missing	1	–	9	–		10	–
Cancer‐related pain							
No pain	21	22.3	356	43.6		377	41.4
Pain	73	77.7	460	56.4	<0.001	533	58.6
Missing	–	–	5	–		5	–
ECOG performance status							
0	16	17.0	389	47.7		405	44.5
1	50	53.2	305	37.4		355	39.0
2–3	28	29.8	122	15.0	<0.001	150	16.5
Missing	–	–	5	–		5	–
Analgesic consumption							
Negligible	21	22.3	414	50.7		435	47.8
≥1	73	77.7	402	49.3	<0.001	475	52.2
Missing	–	–	5	–		5	–
Grade of malignancy							
WHO 1	12	13.3	124	15.5		136	15.3
WHO 2	34	37.8	380	47.5		414	46.5
WHO 3	44	48.9	296	37.0	0.086	340	38.2
Missing	4	–	21	–		25	–
T‐category							
T0–T2	12	13.0	177	21.9		189	21.0
T3–T4	80	87.0	633	78.1	0.067	713	79.0
Missing	2	–	11	–		13	–
PSA, *μ*g/L							
PSA <100	24	25.5	223	27.3		247	27.1
PSA 100–500	39	41.5	353	43.3		392	43.1
PSA >500	31	33.0	240	29.4	0.770	271	29.8
Missing	–	–	5	–		5	–
Soloway score							
1	14	15.1	305	37.8		319	35.4
2–3	79	84.9	502	62.2	<0.001	581	64.6
Missing	1	–	14	–		15	–
Hemoglobin, g/L							
Median, q1–q3	108.5	11.3–129.0	125.0	11.6–141.0	0.004	124.0	11.6–140.0
Missing	0	–	12	–		12	–
SHBG							
Median, q1–q3	47.5	39.8–53.3	39.5	28.0–57.8	0.164	42.0	29.3–57.0
Missing	78	–	711	–		789	–
Estradiol							
Median, q1–q3	64.0	0.1–82.0	65.0	0.2–98.0	0.366	64.5	0.1–96.0
Missing	79	–	712	–		791	–
Testosterone							
Median, q1–q3	12.0	8.3–16.6	13.2	10.0–17.4	0.042	13.0	9.8–17.2
Missing	11	–	79	–		90	–

A total of 94 of 915 (10.4%) patients died of progressive metastatic prostate cancer within 12 months after start of ADT and fulfilled the criteria for early ADT failure. The early ADT failure group had a statistically significant higher burden of cancer‐related pain, a poorer PS, analgesic consumption, and a higher Soloway score compared to the remaining cohort. There were also statistically significant differences with regards to level of hemoglobin and testosterone levels at the time of enrolment between the two groups. Not significantly factors related to early ADT failure were age, treatment, cardiovascular comorbidity, T‐category, grade of malignancy, serum estrogen level, and SHBG at enrolment (Table 1).

Factors prognostically associated with early ADT failure were defined using univariate and multivariate logistic regression analyses (Table 2). Factors significantly associated with early ADT failure in the univariate analyses were as follows: cancer‐related pain, a poor ECOG PS, an analgesic consumption, high Soloway score, and a low level of hemoglobin at enrolment. In the multivariate logistic regression analyses, a poor ECOG PS, analgesic consumption, a high Soloway score, and low levels of hemoglobin were all independent predictors of early ADT failure. Patients with an ECOG PS of 1 or higher, for example, had a threefold likelihood of early ADT failure compared to patients with PS of 0 (Table 2). The results of the 3 scenario analyses did not alter from the base case analyses (the 94 patients who died of progressive metastatic prostate cancer within 12 months), where a poor ECOG PS, analgesic consumption, a high Soloway score, and low levels of hemoglobin were all independent predictors of early ADT failure (Data not shown).

**Table 2:** Univariate and multivariate logistic regression for primary ADT failure

	Univariate		Multivariate	
	OR	CI 95%	OR	CI 95%
Age, years				
<65	1.00	Ref.	–	–
65–74	1.28	0.65–2.78	–	–
≥75	1.73	0.88–3.75	–	–
Initial treatment				
Polyestradiol phosphate	1.00	Ref.	–	–
TAB	1,16	0.75–1.78	–	–
Cardiovascular comorbidity				
None	1.00	Ref.	–	–
Present	1,47	0.75–2.67	–	–
Cancer‐related pain				
No pain	1.00	Ref.	1.00	Ref.
Pain	2.69	1.65–4.56	0.53	0.22–1.27
ECOG Performance status				
0	1.00	Ref.	1.00	Ref.
1	3.99	2.28–7.35	3.09	1.65–6.09
2–3	5.58	2.96–10.88	3.47	1.67–7.44
Analgesic consumption				
Negligible	1.00	Ref.	1.00	Ref.
≥1	3.58	2.20–6.06	2.92	1.25–7.22
Grade of malignancy				
WHO 1	1.00	Ref.	–	–
WHO 2	0.92	0.48–1.91	–	–
WHO 3	1.54	0.81–3.13	–	–
T‐category				
T0–T2	1.00	Ref.	1.00	Ref.
T3–T4	1.86	1.03–3.67	1.80	0.95–3.73
PSA, *μ*g/L				
PSA <100	1.00	Ref.	–	–
PSA 100–500	1.03	0.61–1.77	–	–
PSA >500	1.20	0.69–2.13	–	–
Soloway score				
1	1.00	Ref.	1.00	Ref.
2–3	3.43	1.97–6.41	2.22	1.24–4.25
Hemoglobin, g/L				
High	1.00	Ref.	1.00	Ref.
Low	2.11	1.35–3.35	1.65	1.03–2.69
SHBG				
Low	1.00	Ref.	–	–
High	1.43	0.50–4.27	–	–
Estradiol				
Low	1.00	Ref.	–	–
High	0.86	0.28–2.55	–	–
Testosterone				
Low	1.00	Ref.	–	–
High	0.72	0.45–1.13	–	–

## Discussion

One in ten men with metastatic prostate cancer primarily fails to respond to ADT. In this study we defined a sample of men with prostate cancer and bone metastases as being nonresponders to ADT based on the fact that they died of the disease within 12 months after start of ADT. Nonresponders had a significantly poorer ECOG PS, analgesic consumption, lower hemoglobin, and greater extent of bone metastases. Although it was already realized at the beginning of the ADT‐era that there was a subgroup of prostate cancer patients, who do not primarily responded to ADT, few studies have been undertaken to elucidate this phenomenon even though it continues to be mentioned in the uro‐oncologic literature 1, 4, 5, 6, 7, 10, 11, 36, 37.

A state of castration resistance usually develops after long‐term ADT. Improving the results of metastatic castration‐resistant prostate cancer (mCRPC) treatment is currently regarded as one of the most critical goals in the management of the disease 12, 38, 39, 40. A better understanding of the mechanisms behind secondary resistance following long‐term ADT may eventually result in a better understanding of early ADT failure. However, mCRPC following long‐term ADT is probably a much more heterogeneous condition than metastatic prostate cancer primarily failing to respond to ADT 17.

All men who died of progressive prostate cancer within 12 months of starting ADT were included, though there was a slight bias in the criteria in that any disease‐specific death during the first 12 months was considered to be early ADT failure. There may also have been men who did not respond to ADT that died after 12 months of treatment as well. In the pre‐ADT era, prostate cancer with bone metastases had a very short survival expectation, with a nine‐month mortality rate of 66%. However, long‐term survival was sometimes seen even in the pre‐ADT era 41.

When the idea of ADT evolved, it was suggested that androgen regulation played a central role. When assessing early “failure cases” 42, it was found that those with small testes at time of castration had a poor prognosis. Later, Houghton et al. reported no influence of the pretreatment testosterone concentrations on the outcome of prostate cancer 25. In some studies, however, prostate cancer has been considered to be more aggressive in the presence of low testosterone levels at start of ADT 43, 44.

Medical castration achieved by administration of long‐acting LHRH agonists or estrogens has been demonstrated to be as effective as surgical castration. Although LHRH agonists formulations may differ from estrogens in their suppression of testosterone, there are no data relating these differences to cause‐specific survival. Furthermore, there is no generally accepted limit for serum testosterone after ADT. Recently a value of 0.2 ng/mL or 0.69 nmol/L was suggested, but studies comparing clinical outcome with different castration limits are lacking 45. Uncertainties concerning the role of circulating sex hormones, and response to ADT have prompted the need to measure intraprostatic hormone levels and to compare these with their circulating correlates 26, 46, 47. Residual androgens at levels sufficient to activate the androgen receptor were found in prostate tissue, despite castrate levels of serum testosterone 48.

The previous findings of PSA as prognostic marker for prostate cancer treatment 49 could not be confirmed in this study. The first clinical indication that a patient responds to ADT is that the serum PSA level decreases. In 856 of the originally 915 cases, the serum PSA level had fallen 3 months after start of ADT. The observed PSA decrease in these cases was probably not only due to regression of tumor volume. At the cellular level a fall in PSA levels reflects a decrease in androgen receptor stimulation corresponding to a decreased transcription of the androgen‐responsive PSA gene and secretion of its encoded protein. The reason for the decrease in PSA may, to some extent, be due to the elimination of stimulation of PSA production by circulating androgens 50. We have not assessed the role of PSA kinetics, but focused on patients' variables before treatment start. The reason was the small number of patients in the early ADT failure group. Seven of the 94 patients already died within 3 months and 44 within 9 months. A previous study found that the level of pretreatment PSA or Gleason score at diagnosis could not predict patients likely to be unresponsive to ADT 51. 52, however, demonstrated a correlation between high Gleason score and positivity for chromogranin A in prostate cancer bone metastases and with poor outcome.

Our finding that prostate cancer with more extensive bone metastases is associated with ADT failure agrees with the results of other studies 53. Similarly, poor PS 43, 54, 55, and analgesic consumption 56, have been shown to be predictive for short survival in some studies. Analyses within groups having pain versus no pain at entry revealed poor survival when pain initially was present 5.

In this study all patients had bone metastases, but the presence of distant metastases at other sites was not explored. There is some evidence from clinical trials that treatment results are not related to the site of distant metastases 57. Recently, however, it was shown that the pattern of spread of distant metastases affects prognosis 58. These data, however, require validation.

The negative influence of low hemoglobin at the start of ADT seen in this study is in accordance with previous trials 54, 56.

## Conclusion

Using data from a previous randomized clinical trial, we analyzed and compared the clinical characteristics of men with prostate cancer with bone metastases receiving ADT, and who showed early treatment failure. Using multivariate analyses, poor ECOG PS, analgesic consumption, high Solowey score, and a low hemoglobin were found to be predictive of early ADT failure. Men with these characteristics should be considered for alternative treatment.

## Conflict of Interest

The authors declare no conflict of interest.
